# Optimizing Cosmetic Results in Dental Fluorosis: A Case Report of Bleaching and Microabrasion Techniques

**DOI:** 10.1155/crid/9972925

**Published:** 2025-06-12

**Authors:** Priyanka Bhojwani, Anuja Ikhar, Aditya Patel, Manoj Chandak, Shweta Sedani, Jay Bhoptakar, Khyati Manik, Bhuvaneshwari Khanadali

**Affiliations:** Department of Conservative Dentistry and Endodontics, Sharad Pawar Dental College, Datta Meghe Institute of Higher Education and Research, Wardha, Maharashtra, India

**Keywords:** abrasives, fluoride, hydrochloric acid, phosphoric acid, pumice, sensitivity

## Abstract

Dental discoloration, particularly due to fluorosis, presents a significant aesthetic challenge for many patients. This article explores the combined use of dental bleaching and enamel microabrasion as effective treatments for managing fluorosis-induced discoloration. Dental bleaching, a widely used method for lightening extrinsic and intrinsic stains, has shown promising results in improving the overall appearance of discolored teeth. Enamel microabrasion, which involves the mechanical removal of superficial enamel stains, can enhance the effectiveness of bleaching by addressing more severe or resistant discoloration. The article reviews the mechanisms of these treatments, their clinical indications, and their outcomes in patients with varying degrees of dental fluorosis. Additionally, potential side effects, such as tooth sensitivity and enamel damage, are discussed, emphasizing strategies to minimize these risks. By combining these methods, clinicians can offer more comprehensive, individualized care for patients seeking cosmetic improvements for fluorotic teeth, with attention to both aesthetic outcomes and long-term dental health.

## 1. Introduction

Only the outermost layers of the enamel surface can be treated by enamel microabrasion, which can also remove some intrinsic stains and surface imperfections [1]. Isolated white or yellow patches are frequently observed on an otherwise healthy enamel surface [2–4]. These aesthetic issues can now be resolved thanks to developments in materials and methods that either cover up or eliminate these discolorations [3, 5–7]. Although there is a growing need for aesthetically pleasing smiles, patients' treatment decisions are also greatly influenced by economic considerations. Conservative treatment methods like bleaching, microabrasion, and composite resin restorations are frequently used since they are less expensive and time-consuming. A combination chemomechanical method for cosmetic control of surface enamel flaws, such as staining, hypomineralization, or hypermineralization, can all cause enamel discoloration. For aesthetic improvement, the enamel microabrasion method is frequently suggested. It usually involves a mixture of pumice and 18% hydrochloric acid [8] or 6.6% and 10% hydrochloric acid with silica carbide particles or even 37% phosphoric acid (H_3_PO_4_) gels, employed in equal volume proportions with extrafine grain pumice.

Most dentists are familiar with H_3_PO_4_, a well-known acid frequently used for etching in dental practice. He successfully treated enamel fluorosis without destroying or damaging the enamel by administering hydrochloric acid (HCl) to the maxillary anterior teeth while they were exposed to an alcohol torch flame. For more than 60 years, clinicians mostly avoided the method despite these encouraging outcomes because of worries about possible damage to the enamel. McCloskey did not start using acid and pumice together until 1984 [8], and Croll first used the term “microabrasion” to refer to this method in 1986 [9].

## 2. Diagnosis and Etiology

**Figure 1:**
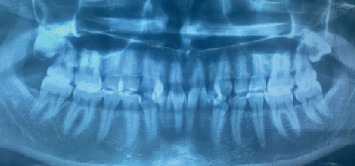
OPG showing the complete upper and lower arches.

A 28-year-old female patient presented to the outpatient department of Sharad Pawar Dental College and Hospital with a chief complaint of dental fluorosis. An orthopantomogram (OPG) radiograph was taken to assess any additional diagnostic findings, as shown in Figure 1.

**Figure 2:**
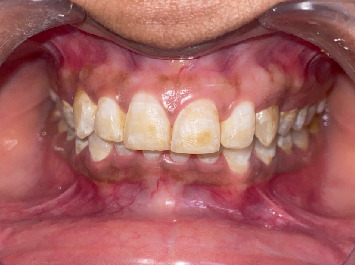
Preoperative clinical images.

**Figure 3:**
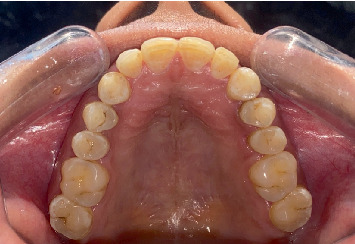
Preoperative clinical images of the upper arch.

**Figure 4:**
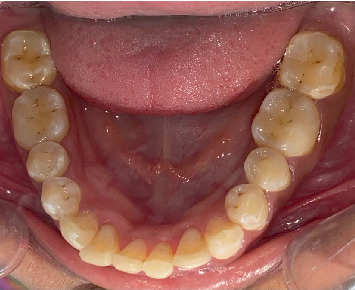
Preoperative clinical images of the lower arch.

Preoperative clinical images were recorded as shown in Figures 2, 3, and 4.

The final diagnosis was generalized dental fluorosis. The patient was provided with counseling, and the treatment plan was thoroughly explained.

## 3. Treatment Procedure

OptraDam was applied to the teeth, and 37% H_3_PO_4_ was selected for microabrasion, as shown in Figure 3. Following the application of OptraDam (OptraDam Plus, Ivoclar Vivadent, Gurugram 122011, Haryana India), floss knots were placed to ensure proper retention throughout the procedure. Microabrasion using 37% H_3_PO_4_ (Prime Dental, Thane, Maharashtra, India) and pumice (Vishal Dentocare Plak-Check Ahmedabad, Gujarat, India) powder was performed three times on each of the upper anterior teeth, from canine to canine, with 60-s intervals between each application.

The patient was prescribed Thermoseal RA (ICPA Health Products Limited Ankleshwar, Gujarat, India) to manage sensitivity, and postoperative instructions were provided.

## 4. Treatment Progress

**Figure 5:**
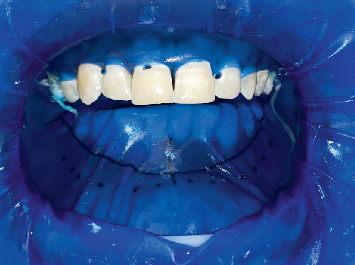
Bleaching process.

Fifteen days later, the patient was recalled. OptraDam and a gingival barrier (ICPA Health Products Limited Ankleshwar, Gujarat, India) were reapplied to protect the gingiva from the caustic effects of bleaching agents. For bleaching, a hydrogen peroxide bleaching kit (SDI Pola Office, Bayswater, Victoria, Australia), consisting of a powder and liquid, was used. The powder and liquid were mixed and applied to the tooth surfaces using a wooden applicator. The bleaching process was accelerated using a dental LED (Philips Zoom WhiteSpeed, United States) bleaching device, as shown in Figure 5. The patient was again prescribed Thermoseal RA (ICPA RA Thermoseal Toothpaste) for sensitivity, and postoperative instructions were reiterated.

## 5. Treatment Alternatives

Treatment options, including veneers, crowns, and macroabrasion, were discussed with the patient and his parents. Ultimately, the enamel microabrasion technique was selected to address the stains and pitted areas on the facial surfaces of the upper incisors and canines.

## 6. Treatment Result Case Report

**Figure 6:**
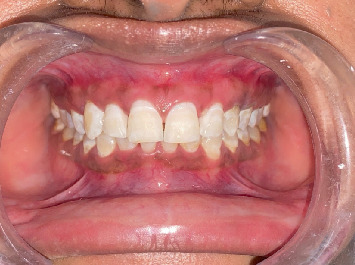
Postoperative results.

The next day, the patient was recalled for postoperative evaluation as shown in Figure 6. Telephonic communication was initiated to assess the severity of the sensitivity experienced by the patient. The patient reported experiencing sensitivity during the procedure, which persisted for 2 days afterward.

Table 1 shows the checklist of the procedures performed.

## 7. Discussion

Teeth treated with microabrasion exhibit a smooth, glossy, glass-like enamel surface. However, excessive fluoride intake during the years of enamel formation (amelogenesis) can result in discoloration of the outer enamel layer, with shades ranging from white to brown [10]. Some of these discolorations are referred to as “idiopathic enamel demineralization” [11].

Various clinical techniques are available to enhance the aesthetic appearance of affected teeth, including tooth whitening, enamel microabrasion, and restorative approaches using tooth-colored adhesive materials [12]. Research indicates that enamel microabrasion, which involves the use of acidic and abrasive agents, results in minimal and nearly imperceptible enamel loss while delivering rapid and lasting cosmetic improvements [13].

The key indicator of treatment success is the elimination or reduction of surface stains. To optimize visual outcomes, microabrasion is often combined with complementary procedures such as bleaching or adhesive restorations [11]. It is recommended to delay bleaching for several weeks after completing the microabrasion process to ensure enamel recovery and stability.

However, microabrasion is ineffective when discoloration originates from the dentin, such as in cases of tetracycline staining or dentinogenesis imperfecta. Additionally, it may be contraindicated or deferred in patients who exhibit poor lip seal. In such cases, the lack of natural lip coverage prevents the formation of a protective salivary pellicle on the enamel, resulting in persistently dry enamel surfaces. This dryness can intensify the visibility of demineralization spots, reducing the effectiveness of cosmetic treatments.

Sundfeld et al. noted that, in certain cases, effective stain removal may require interventions such as lip repositioning or other corrective techniques to adjust lip placement [11]. In some instances, addressing issues related to lip alignment through orthodontic treatment or speech therapy (phonoaudiology) may be necessary prior to initiating enamel microabrasion [3, 14–16].

It is also important to consider that immediately following dental bleaching procedures, white stains on the teeth may temporarily appear more prominent due to dehydration. However, these stains typically diminish within a few days as the enamel rehydrates through contact with saliva.

To effectively remove enamel stains with minimal surface loss, Cavanaugh and Sundfeld et al. recommended using a compound containing 18% hydrochloric acid combined with pumice [3, 6]. Compared to a mixture of H_3_PO_4_ and pumice, the hydrochloric acid–based approach required significantly less treatment time [5].

Microabrasion produces a glossy, prism-free enamel surface that continues to improve in appearance over time [17]. This enhancement is attributed to what has been described as the “abrosion effect”—a combination of abrasion and erosion—which results in mineral compaction at the enamel surface. In a 2011 study, Fragoso et al. found that posttreatment polishing with either diamond paste or fluoride prophylactic paste enhanced enamel hardness and surface smoothness [18]. The observed increase in microhardness may be due to the compressive effects of micronized diamond particles in the diamond paste and the densely packed calcium carbonate, pumice, and fluoride in the prophylactic paste.

In an in vitro study conducted by Segura et al. [18], enamel surfaces treated with microabrasion demonstrated greater resistance to demineralization compared to untreated enamel, as observed under polarized light microscopy. This enhanced resistance is likely due to reduced bacterial adherence on the treated surfaces [19].

Emerging adjunctive treatments, including casein phosphopeptide-amorphous calcium phosphate (CPP-ACP) and biomimetic hydroxyapatite, have shown potential in the management of fluorosis. Further studies are warranted to assess the efficacy of these compounds in clinical settings [20, 21].

Conventional approaches for managing severe dental fluorosis, such as crowns, veneers, and composite restorations, are often invasive and necessitate significant tooth structure removal. In contrast, microabrasion offers a conservative, minimally invasive alternative for improving dental aesthetics without extensive enamel loss. Another innovative technique gaining recognition is resin infiltration. In a comparative study by Reddy et al., the combination of 37% H_3_PO_4_ with pumice, Opalustre, and resin infiltration was evaluated. The results showed that the combination treatment yielded a mean surface roughness of 10.42, while resin infiltration alone resulted in significantly lower surface roughness at 1.99—indicating a less invasive approach with minimal posttreatment surface alteration [22].

A noted limitation of the current approach is that resin infiltration might have provided a more aesthetically favorable outcome, producing a glossier, less opaque finish on the treated enamel surfaces.

## 8. Conclusion

When used properly, the microabrasion method can improve the teeth's uniformity in appearance and color, which will help the patient feel better about themselves. Over time, microabrasion has proven to be a safe procedure, yielding lasting improvements in the appearance of patients' smiles.

## Figures and Tables

**Table 1:** Steps of procedure.

**Procedure**	**Description**
Isolation	To achieve an excellent isolation, rubber dam and gingival barrier were used
Microabrasion	Microabrasion was performed using pumice and 37% phosphoric acid
Follow-up	Frequently, patients experience mild sensitivity few hours after microabrasion
Bleaching	Pola Office bleach was used
Follow-up	Frequent telephonic follow-up and occasional clinic visits were performed

## Data Availability

Data sharing is not applicable to this article as no new data were created or analyzed in this study.
